# Beyond the Synapse: *FMR1* and FMRP Molecular Mechanisms in the Nucleus

**DOI:** 10.3390/ijms26010214

**Published:** 2024-12-30

**Authors:** Nicole Hansen, Anna Dischler, Caroline Dias

**Affiliations:** Department of Pediatrics, University of Colorado Anschutz Medical Campus, Aurora, CO 80045, USA; nicole.hansen@cuanschutz.edu (N.H.); anna.dischler@cuanschutz.edu (A.D.)

**Keywords:** Fragile X, FMR1, FMRP, R-loops, chromatin, nucleus

## Abstract

*FMR1* (Fragile X messenger ribonucleoprotein 1), located on the X-chromosome, encodes the multi-functional FMR1 protein (FMRP), critical to brain development and function. Trinucleotide CGG repeat expansions at this locus cause a range of neurological disorders, collectively referred to as Fragile X-related conditions. The most well-known of these is Fragile X syndrome, a neurodevelopmental disorder associated with syndromic facial features, autism, intellectual disabilities, and seizures. However, CGG expansions of different sizes also confer a risk of neuropsychiatric and neurodegenerative disorders throughout the lifespan, through distinct molecular mechanisms. Although Fragile X syndrome is associated with downstream synaptic deficits and neuronal hyperexcitability, work in the past decade has demonstrated that both the causative *FMR1* trinucleotide repeat expansion and FMRP itself play important roles in nuclear function and regulation, including non-canonical nucleic acid structure formation and chromatin dynamics. These effects are critical to cellular pathophysiology, although the full extent of their contribution to clinical phenotypes is only just emerging. Here, we present a focused review on some of the nuclear consequences of *FMR1*/FMRP dysregulation, including parallels in other repeat expansion disorders, ranging from studies in model systems to human cells and tissues.

## 1. Introduction

Fragile X-related conditions include Fragile X syndrome (FXS), Fragile X-associated tremor/ataxia syndrome (FXTAS), and Fragile X-associated neuropsychiatric disorders (FXANDs). FXS is a neurodevelopmental disorder. On the other hand, FXTAS is a late-onset neurodegenerative disorder with clinical features including an ataxic gait, tremor, executive dysfunction, cognitive decline, neuropathy, and mood dysregulation [1,2]. FXTAS affects some carriers of the Fragile X premutation, which is a trinucleotide repeat ranging from ~55–200 CGGs. This CGG repeat expansion is located in the 5′ untranslated region (UTR) of *FMR1* and leads to increased FMR1 mRNA and variably reduced FMR1 protein (FMRP) levels [3,4,5,6]. Additionally, it is now clear that the premutation affords risk for adult-onset psychiatric diagnoses such as depression and anxiety (i.e., FXANDs) [7,8,9,10]. Alternatively, individuals with the full Fragile X mutation (≥200 CGGs) generally develop *FMR1* hypermethylation and transcriptional silencing, leading to the elimination of FMR1 mRNA and FMRP expression, and the neurodevelopmental phenotype of FXS [11,12,13]. However, it is important to note *FMR1* methylation can also present as mosaic, meaning some cells may escape hypermethylation and silencing. In fact, *FMR1* methylation mosaicism has been observed in multiple individuals with FXS. The subsequent transcription of *FMR1* in specific populations of cells leads to an overall increase in FMRP, and these individuals are typically less cognitively affected than those without methylation mosaicism [14,15,16,17]. Individuals with FXS do not develop FXTAS and individuals with FXTAS do not have preceding FXS, implicating distinct molecular mechanisms. Specifically, the unique increases in *FMR1* mRNA in individuals with the premutation has led to multiple mRNA gain-of-function and toxic peptide formation hypotheses [18,19,20]. Although the magnitude of toxic FMR1 mRNA increases seen in the brain are more modest as compared to the peripheral blood and tissue [21], on the other hand, the loss of FMRP in individuals with FXS is known to be causative of synaptic dysfunction and neuronal hyperexcitability. However, exciting work in the past decade has revealed complex nuclear dysregulation in Fragile X-related conditions beyond changes in neuronal synaptic function.

In this review, we highlight recent advances in our understanding of nuclear dysregulation in Fragile X-related conditions and call attention to parallels seen in other repeat expansion disorders. Specifically, we focus on the roles of non-canonical nucleic acid structures, such as R-loops and G-quadruplexes, and types of chromatin organization such as topologically associated domains (TADs) in these conditions. This review also highlights areas that are amenable to future study from both a basic science and therapeutic perspective.

## 2. Nucleic Acid Secondary Structures in Fragile X-Related Conditions

**Figure 1:**
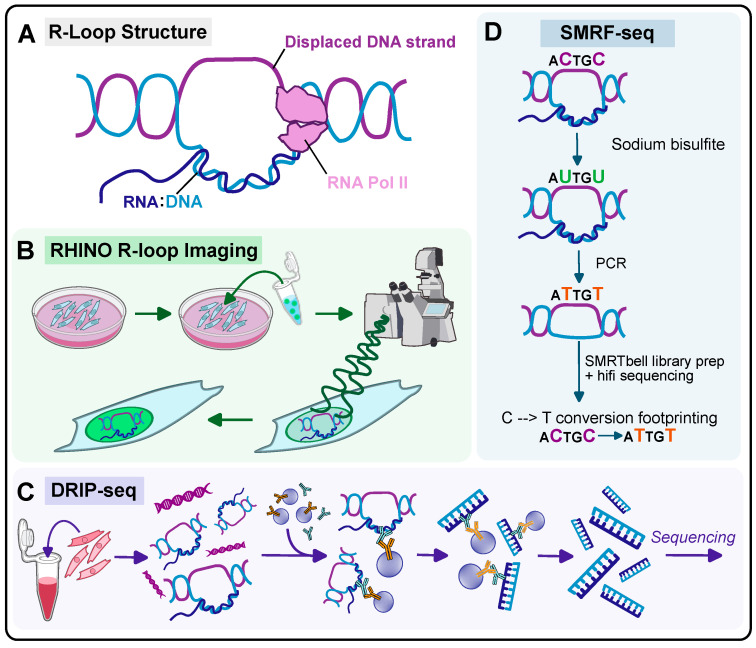
R-loop structure and detection methods. (**A**) Schematic of R-loop structure. (**B**) Imaging R-loops in cultured cells involves transient transfection of RHINO using Lipofectamine 3000. Live cell imaging with a RHINO can be performed with a confocal microscope. (**C**) A diagram showing the DRIP-seq workflow, which involves DNA harvesting and fragmentation, the addition of the S9.6 antibody and conjugated beads, washing, and the sequencing of DNA:RNA hybrids. (**D**) Schematic of SMRF-seq workflow. The input DNA is treated with RNase H1 to digest the associated RNA molecule and create a reference for C-to-T conversion footprinting. Additional DNA is treated with non-denaturing sodium bisulfite to convert available cytosines to uracils. PCR amplification is then conducted to convert uracils to thymines. Then, both the reference and sample libraries are prepared using the PacBio SMRTbell library prep kit and sequenced using PacBio Hifi sequencing. C-to-T conversion footprinting can be conducted by comparing the sample to the reference.

The secondary structure of nucleic acids, the blueprint for DNA and RNA, is an important aspect of nucleic acid function. Beyond simple single-stranded or double-helix structures, complex structural arrangements can arise due to the intrinsic properties of the nucleotide sequence itself, such as hairpins and cloverleaf formations. R-loops are three-stranded DNA-RNA hybrids consisting of an RNA molecule that hybridizes to DNA, simultaneously creating single-stranded DNA through displacement [22,23] (Figure 1A). R-loops play pervasive roles in cellular physiology across the genome [24,25]. R-loops that are created at transcriptional start sites can remain in the transcribed region and promote the formation of other non-canonical nucleic acid structures like G-quadraplexes and hairpins, with the resulting R-loop often being more stable than double-stranded DNA (dsDNA) [26,27]. Although R-loops are naturally occurring and play a role in dynamic gene expression regulation, they can also lead to genome instability [28,29,30,31]. For example, the accumulation of these structures can lead to the stalling of RNA polymerase II and the deterioration of the replication fork, changes in the chromatin structure, and histone modifications [22,32,33]. However, R-loops also serve a protective role, for example, in preventing CpG-rich sequences from acquiring DNA methylation and the subsequent gene silencing [34,35] and regulating transcription termination [36]. Thus, R-loops play both protective and pathogenic roles in the genome structure and integrity depending on the cellular context. In the human brain, there is preliminary evidence that R-loops play a critical role in neural differentiation, cell type-specific transcription, and connectivity [37,38].

Techniques that accurately and sensitively detect R-loops are critical to resolving R-loop biology in Fragile X-related conditions, including their formation and localization (Figure 1B–D). Traditionally, the S9.6 antibody is used in DRIP (DNA-RNA immunoprecipitation) or immunofluorescence to directly recognize DNA-RNA hybrids (Figure 1C). This antibody has played a central role in advancing the understanding of R-loop structure and function. However, cross-reactivity to double-stranded RNA is a potential confounder that must be accounted for experimentally [39]. A catalytically inactive RNase H1 enzyme that has a high affinity for RNA–DNA hybrids has also been applied to study R-loops. Additionally, techniques such as those using a RHINO (**R**NA-DNA **h**ybrid-b**in**ding sens**o**r) and SMRF-seq (single-molecule R-loop footprinting coupled with PacBio sequencing) have also emerged more recently on the cutting edge of R-loop detection (Figure 1B,D) [40,41]. SMRF-seq leverages the bisulfite-based deamination of exposed cytosines to uracils on the unpaired DNA strand, then proceeds with long-read sequencing to identify these genomic ‘tags’ at a single-molecule resolution (Figure 1D). RHINO is a genetically encoded sensor that can be used to detect DNA-RNA hybrids in live cell imaging systems and has been applied in cellular model systems such as human osteosarcoma cells (U2OS) (Figure 1B) [42]. Given these advances, we can now obtain detailed information on R-loop formation within the nucleus at an unprecedented scale and precision [28,42,43].

R-loop formation likely plays a role at multiple levels in Fragile X-related conditions and may depend on the proliferation status of the cell type. Due to the repetitive nature of the sequence and its high GC content, the 5′ UTR of *FMR1* is especially prone to forming R-loops even at the ‘normal’ length, likely related to R-loop physiological roles in transcriptional regulation [34,35]. Diab and colleagues confirmed enrichment for R-loops in Fragile X-expanded, unmethylated alleles when compared to wild-type alleles using DRIP analysis [27]. Furthermore, in FXS patient cells, it was shown that FXS patient cells accumulated evidence of DNA damage in R-loop-forming regions. There was also evidence of an increased S9.6 signal in these cells when placed under replication stress [44]. Because exogenous FMRP expression ameliorated the R-loop-associated DNA damage, the authors proposed that FMRP is directly involved in genome integrity and R-loop regulation and specifically speculated that this may contribute to symptoms of an intellectual disability due to alterations during neurogenesis. Work in human fibroblast premutation cell lines has separately demonstrated increased R-loop formation [34]. Thus, there is evidence in both FXS and FXTAS of marked R-loop dysregulation.

Additional studies have put forth evidence that FMRP directly regulates R-loop accumulation, with some evidence that FMRP binds R-loops [45,46]. How might FMRP regulate R-loop formation in the nucleus? Although FMRP is traditionally considered predominantly cytoplasmic, there is some evidence that it is involved in nucleocytoplasmic shuttling and nuclear regulation [47,48]. Chakraborty and colleagues found that FMRP directly interacts with the RNA helicase DHX9 and proposed that this could contribute to resolving R-loops [45]. Liquid–liquid phase separation (LLPS) is a mechanism by which membrane-less compartments form through weak interactions, driving the organization of biological activities. There has been some evidence that these structures may also modulate genomic regulation and integrity [49,50]. Preliminary work has suggested that FMRP can form LLPS droplets with synthetic R-loops within the nucleus [51]. Together, these suggest FMRP’s direct involvement in the R-loop surveillance pathway [44]. Given that the *FMR1* premutation and full mutation are known to be associated with lower to nonexistent concentrations of FMRP [11,52], reduced FMRP could directly contribute to the R-loop dysregulation through these or other novel mechanisms. It will be important to further characterize FMRP’s intranuclear role and binding dynamics with R-loops in tissue or cells derived from FXS or FXTAS patients with orthogonal methods to replicate prior findings regarding the nuclear FMRP function and identify novel biology. For example, exploring the differences between brain cell types, including in both proliferating and non-proliferating contexts, in terms of R-loop formation and maintenance in the human condition could reveal novel therapeutic approaches.

In fact, recent work has already identified R-loops as a potential therapeutic strategy in Fragile X-related conditions [53]. Lee and colleagues used CpG demethylation to identify a positive feedback loop of R-loop formation and DNA repair that led to the contraction of >200 CGG expansions, independent of artificial gene editing approaches. However, it should be noted that their demethylation approach did not seem able to contract below the premutation range to a totally ‘normal’ repeat size, suggesting (1) distinct mechanisms in premutation and full mutation expansions and (2) that this approach may not generate truly ‘normal’ repeats, critical in considering therapeutic development given that the premutation allele retains a significant risk for neurological dysfunction. Other therapeutic approaches include antisense oligonucleotide (ASO) therapy to treat R-loop accumulation in FXTAS. Derbis and colleagues demonstrated that ASO treatment was shown to impact R-loops in fibroblasts derived from patients with FXTAS. The proposed mechanism included altering the thermodynamic stability of the R-loops produced during *FMR1* transcription, and the binding of the ASO disrupts this thermodynamic stability [54]. These investigators also demonstrated that ASO use in a mouse model of FXTAS rescued behavioral and cellular phenotypes. Given the interest in ASO therapy in other repeat expansion disorders, it will be important to unravel the role of R-loops more universally in the mediating effects of these therapies.

Additional nucleic acid structures implicated in Fragile X conditions are G-quadruplexes, nucleic acid secondary structures formed in guanine-rich sequences. G-quadruplexes are crucial for gene regulation and the proper localization of transcripts and are enriched in regulatory regions, including in the 5′ or 3′ untranslated regions [55,56,57,58,59,60]. Additionally, they are involved in epigenetic regulation by modifying histones and methylating cytosines to regulate chromatin dynamics [61,62,63]. To understand the effect that G-quadruplexes in the 5′ UTR of FMR1 mRNA have on subcellular localization, Sirois et al. used an in silico model and predicted both the formation of G-quadruplexes in the 5′ UTR of FMR1 and a loss of said G-quadruplex structures upon the deletion of the CGG tract. They further showed that the destabilization of the CGG repeat tract disrupted the subcellular localization of FMR1 mRNA, and therefore FMRP, by deleting the CGG motif from the 5′ UTR of FMR1 mRNA (zero CGGs). The 0 CGG construct caused the premature localization of FMR1 mRNA to dendrites when compared to the modal length construct (31 CGGs), inadvertently increasing FMRP in the cell soma and leading to further downstream effects [64]. They concluded that the neuronal subcellular localization of FMR1 mRNA may be mediated by the G-quadruplex structure within the transcript itself.

To gain insight into the predicted G-quadruplex landscape in FMR1 mRNA, we used quadruplex-forming G-rich sequences (QGRSs), a G-quadruplex analysis tool, and focused on the 5′ UTR and exon 1 of FMR1 mRNA [65]. QGRSs detected 10 unique quadruplex-forming G-rich sequences in the canonical (19 CGG) mRNA. An intermediate repeat (52 CGGs) contained 14 quadruplex-forming sequences. Upon the expansion of *FMR1* into the premutation range, the presence of these sequences increased. A premutation allele with 117 CGG repeats displayed 21 unique quadruplex-forming sequences. However, when using QGRSs to predict G-quadruplexes within the 5′ UTR and exon 1 of the artificial construct (31 CGGs) created by Sirois and colleagues, they were only able to detect nine quadruplex-forming sequences. This construct did not contain AGG insertions within the CGG repeat region, sequence changes that are known to impact the repeat stability [66,67] (Supplementary Figure S1). Given the localization differences seen between the 0 CGG construct and the 31 CGG construct described above, it would be interesting to look further into mRNA trafficking differences even between ‘normal’ and intermediate transcripts, as they differ by ~4 quadruplex forming regions (Supplementary Figure S1).

However, it is important to note that the deletion of the CGG repeat tract may affect more than the native G-quadruplex structures located within it. Additionally, previous characterizations of the CGG repeat tract seemed to focus on the CGG motif alone, as opposed to the CGG repeat tract within the context of FMR1 mRNA. These studies highlight the importance of studying not only pathogenic CGG repeat expansions but also the endogenous role of this motif in the ‘normal’ length to further elucidate *FMR1*’s complex molecular mechanisms. Additionally, there is evidence that FMRP directly interacts with FMR1 mRNA through additional G-quadruplexes at the FMRP binding site, and that this may contribute to the regulation of alternative splicing [68]. Upon the creation of a mutant *FMR1* line that abolished both G-quadruplexes at the FMRP binding site on FMR1 mRNA, Didiot and colleagues observed dramatically reduced FMRP binding. However, they did not see changes to FMR1 mRNA translation or localization, potentially suggesting that G-quadruplexes in the FMRP binding site play more of a role in the regulation of splicing than in transport [68]. Immunoprecipitation studies of FMRP-RNA complexes revealed that many FMRP-bound mRNAs contain G-quadruplexes [69], implicating them more generally in Fragile X conditions.

Thus, alterations in nucleic acid structural regulation seem to occur at multiple levels in Fragile X-related conditions, with differences related to the length of the CGG expansion and the level of FMRP, as well as the cell type-specific context.

## 3. R-Loop Dysfunction in Other Disorders

A growing body of literature supports R-loop dysregulation in neurologic disorders beyond FXS and FXTAS. Repeat expansion disorders such as Friedreich ataxia and amyotrophic lateral sclerosis (ALS) have also been implicated in R-loop dysregulation [70]. ALS is a neurodegenerative disorder with both sporadic and inherited etiologies [71]. A common genetic cause of both ALS and frontotemporal dementia is a repeat expansion in the first intron of *C9ORF72* [72,73,74]. These mutations have been associated with higher rates of R-loop formation. Additionally, TAR DNA binding protein 43 (TDP-43) is found in neuronal inclusions in >97% of patients [75,76]. TDP-43 is a DNA/RNA binding protein encoded by *TARDBP*, which regulates transcription, and is involved in RNA splicing and processing. TDP-43 prevents both R-loop accumulation and DNA breaks in neuronal and non-neuronal cells [77]. Mutations to TDP-43 cause R-loop accumulation in neuroblast-like cells, and the silencing of TDP-43 using siRNA increases the S9.6 antibody signal significantly, further demonstrating TDP-43’s role in R-loop maintenance [78,79,80]. Interestingly, TDP-43 acts with FMRP to translationally regulate FMRP target mRNAs and has been found to co-localize with FMRP in primary mouse cortical neuronal cells [81,82,83]. Neurologic dysfunction related to R-loop dysregulation is not limited to repeat expansion disorders. For example, mutations in *SETX*, encoding a helicase involved in R-loop regulation, lead to multiple neurologic phenotypes [84]. However, overall, there is limited primary research into the relationship between R-loop accumulation and how this cellular pathophysiology links to the clinical spectrum of neurological disease more broadly. This is critical given some conflicting findings. For example, researchers identified tissue-specific R-loop accumulation that was not observed in the brain in their mouse model of *Setx* dysfunction and other autosomal recessive ataxias [85]. Whether this represents true tissue specificity present in the human condition or species-level differences requires further investigation with additional cross-species comparisons and the direct study of human cellular models and tissues.

## 4. Chromatin Structure and Organization in Fragile X-Related Conditions

Topologically associating domains (TADs) are large-scale genomic sections that have high rates of self-interaction [86,87]. Identified over 10 years ago, TADs have since been implicated in chromatin folding, reorganization, and in preventing the spread of heterochromatin [87,88]. Additionally, TADs are stable through cell divisions, are evolutionarily conserved, and are enriched for transcription start sites and housekeeping genes [87,89,90,91,92,93,94]. The regions between TADs, termed TAD boundaries, are highly transcribed and enriched for CCCTC binding factor (CTCF) sites, with CTCF being a multi-functional regulatory transcription factor. The disruption of CTCF sites triggers the disruption of TAD boundaries, and mutations of CTCF/cohesion-binding motifs are frequently observed in many cancers [87,95,96,97,98,99]. Techniques capable of visualizing TADs have provided insight into TAD function and regulation. Approaches that capture chromatin conformations (chromosome capture techniques) include 3C, 4C, 5C, and Hi-C and generally use the physical cross-linking of chromatin to identify physically proximal sections at varying levels of resolution (targeted vs. genome-wide, etc.).

Chromatin conformation profiling has revealed that repeat expansions may be intimately linked to TADs. In a transformative paper, Sun and colleagues demonstrated that disease-associated repeat expansion genes are spatially placed at the boundaries between chromatin domains, including *FMR1*, *ATXN7*, and *HTT* [100]. This work identified the large-scale reorganization of the chromatin landscape in FXS [100]. Additionally, FXS patient-derived iPSC-NPC lines displayed the misfolding of TADs, subTADs, and the disruption of the *FMR1* TAD boundary [101]. Topological disruption around the *FMR1* locus was also observed in FXS patient B cells and fibroblasts, demonstrating the remarkable conservation of the phenomenon across cell types [100,101]. Because *FMR1* itself has several CTCF binding sites, TAD boundary disruption also leads to the loss of CTCF occupancy [102,103]. The subsequent loss of CTCF at *FMR1* is correlated with the degree of *FMR1* silencing [100]. Exploration into the TAD-related epigenetic mechanisms of repeat expansion disorders beyond FXS and FXTAS is minimal, despite several pathogenic short tandem repeats altering DNA methylation, which could directly affect CTCF binding sites and TADs [104].

**Figure 2:**
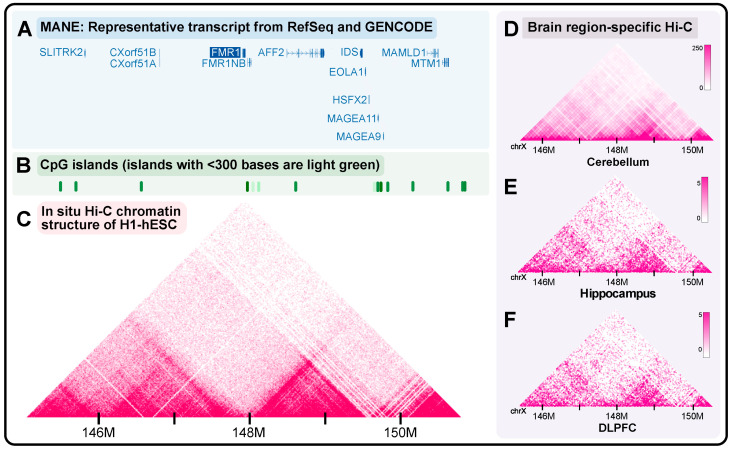
Chromatin interaction patterns at the *FMR1* locus. (**A**) Schematic of the genes upstream and downstream of the FMR1 locus. (**B**) CpG islands around the *FMR1* locus. (**C**) The Hi-C of H1-hESCs maps chromatin interactions around the *FMR1* locus. Figure generated using the Hi-C and Micro-C track in the UCSC genome browser (subtrack: in situ Hi-C chromatin structure of H1-ESC). (**D**) Hi-C of human cerebellum at 100 kb resolution. (**E**) Hi-C of human hippocampus at 40 kb resolution. (**F**) Hi-C of human dorsolateral prefrontal cortex at 40 kb resolution. Figures were generated using the UCSC genome browser and Hi-C data browser (hg38).

To explore the native chromatin structure around *FMR1*, we utilized the UCSC genome browser to visualize the broader *FMR1* locus in human embryonic stem cells (H1-hESCs) [105], including neighboring CpG islands (Figure 2A–C). Prior work has demonstrated that H3K9me3 in several FXS iPSC-NPC lines spans an additional gene involved in synaptic maintenance, SLITRK2 [106,107]. While ‘normal’-length and premutation-length iPSC-NPCs showed FMR1 looping to SLITRK2 directly, this interaction was abolished in full mutation-length iPSC-NPCs [101]. As a result, there was a decrease in SLITRK2 mRNA in FXS cell lines. We also used the Hi-C data browser, a resource made available by the Yue lab to demonstrate brain region-specific maps of the chromatin structure [108]. We generated Hi-C profiles specific to the human cerebellum (Figure 2D) [109], hippocampus (Figure 2E), and dorsolateral prefrontal cortex (Figure 2F) [110]. Hi-C maps displayed similarities regardless of the brain region. Although for the ease of conceptualization, we presented the nucleic acid structure and chromatin organization separately, these elements influence each other. For example, TAD boundaries with G-quadruplexes have been shown to interact more frequently and contain more CTCF [111,112]. R-loops have also been shown to be critical in TAD formation in multiple contexts [113,114]. Still, open questions remain as to whether there is a causal relationship between the location of disease-associated repeats and chromatin boundaries, as well as regarding the potential intersection of R-loops and G-quadruplexes in maintaining the TAD landscape in the context of Fragile X-related conditions.

**Figure 3:**
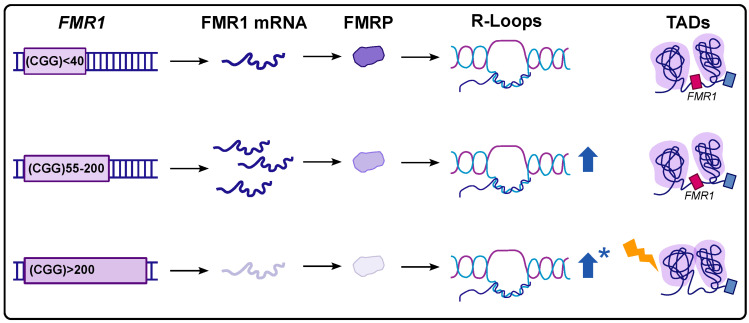
Nuclear dysregulation in Fragile X-related conditions. Alterations in R-loop and TAD formation in typical-length, premutation-length, and full mutation-length alleles. * Note that R-loop dysregulation in Fragile X syndrome can depend on cellular context, for example, demethylation, replication stress, and DNA damage.

The above work made it clear that FXS disrupts the 3D chromatin organization in human cells, but is this disruption detected in FXTAS? Recent work has applied CRISPR engineering to “cut-back” the CGG repeat tract to the premutation (100–199 CGG) repeat range. The Hi-C analysis of these premutation clones demonstrated that TAD boundaries had been reestablished at the broader *FMR1* locus in FXTAS patient cell lines (Figure 3) [101]. In fact, FXTAS Hi-C profiles more closely resembled those of control individuals than the profiles of those with FXS. Interestingly, they did find that large-scale inter-chromosomal interactions impacted the global genome instability, including the heterochromatinization of long synaptic genes. These results demonstrate marked differences in the spatial epigenetic landscape of FXS and FXTAS. Because the brain is so heterogenous, future work on developing methods to determine the cell type-specific epigenetic profiles in FXS and FXTAS will be important. For example, are the effects on TAD disruption truly homogenous, or is there a specific population of cells that demonstrates an increased burden of TAD boundary disruption or, conversely, a population of cells that preserve “typical” TAD boundaries within the human brain? Does the degree of TAD boundary ablation correlate with either the *FMR1* CGG repeat length or *FMR1* methylation and is one more important than the other? Finally, an interesting observation noted by the group is that despite screening over 900 clones with their CRISPR strategy to contract the CGG expansion, they obtained only seven premutation clones and zero ‘normal’ clones. This highlights the significant technical difficulties of working with the CGG repeat in cloning and gene editing, difficulties that remain a major barrier in the field today. Thus, it is possible that a better understanding of the mechanisms driving chromatin dysregulation in Fragile X-related conditions could thus also pave the way for technical progress in basic science approaches and gene editing.

## 5. Conclusions

In this review, we have discussed the considerable complexities in nuclear dysregulation related to *FMR1*, ranging from local nucleic acid structure formation to long-range chromatin interactions. The dysregulation of nuclear regulation and organization is a critical feature of Fragile X-related conditions, occurring at multiple levels, including at the DNA (the CGG locus), RNA (FMR1 mRNA), and protein (FMRP) levels (Figure 3). A better understanding of this dysregulation in clinical phenotypes is a critical area for future study. For example, are there specific clinical phenotypes that may be related specifically to nuclear dysfunction? And importantly, is part of the frustration with clinical trial progress in Fragile X-related conditions related to ongoing nuclear dysfunction, which is not captured well in animal models, nor currently targeted in therapeutic development? Additionally, careful experiments which selectively parse out the individual role of the (1) repeat expansion in DNA, (2) the FMR1 mRNA transcript, and (3) FMRP itself will be critical given the complex relationships described here, with the cellular and developmental context being a key consideration, for example, regarding proliferating progenitors in neurodevelopment vs. post-mitotic neurons in the aging brain. Finally, the role of this dysregulation in other related conditions, such as FXANDs, or other neurological conditions is an area ripe for further study. Recent advances in high-resolution in situ and sequencing technologies will allow for the rapid acceleration of our understanding of this fascinating aspect of Fragile X-related conditions. 

## Supplementary Materials

The following supporting information can be downloaded at https://www.mdpi.com/article/10.3390/ijms26010214/s1.
