# Supplementary material for: Beyond the Synapse: FMR1 and FMRP Molecular Mechanisms in the Nucleus
Source: Int J Mol Sci. 2024 Dec 30;26(1):214. doi: 10.3390/ijms26010214 (PMC11720320; doi:10.3390/ijms26010214)
Supplement: Supplementary file 1 [file ijms-26-00214-s001.zip › ijms-3357404-supplementary.pdf]

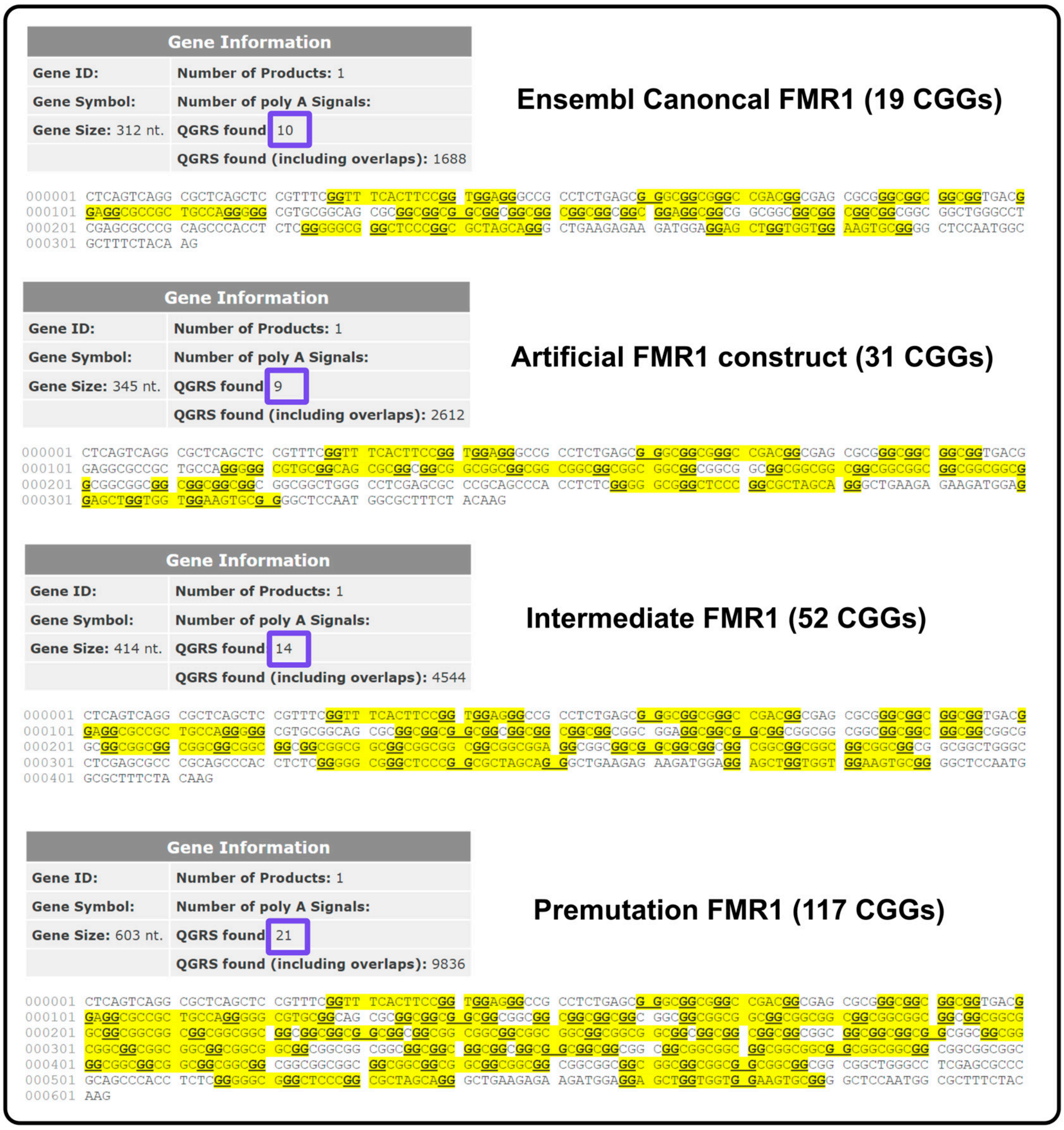

Figure S1. Prediction of G-quadruplex structure in varying CGG motif sizes. Purple box indicates predicted number of g-quadruplex forming G-rich sequences depending on CGG length as described in main text.
